# Higher miR‐543 levels correlate with lower *STK31* expression and longer pancreatic cancer survival

**DOI:** 10.1002/cam4.3559

**Published:** 2020-10-30

**Authors:** Weizhong Yuan, Hao Gao, Guangfu Wang, Yi Miao, Kuirong Jiang, Kai Zhang, Junli Wu

**Affiliations:** ^1^ Pancreatic Center & Department of General Surgery The First Affiliated Hospital of Nanjing Medical University Nanjing Jiangsu China; ^2^ Pancreas Institute of Nanjing Medical University Nanjing Jiangsu China; ^3^ Department of General Surgery Nanjing Meishan Hospital Nanjing Jiangsu China

**Keywords:** miR‐543, Pancreatic cancer, *STK31*, Survival

## Abstract

Pancreatic cancer (PC) is one of the most malignant gastrointestinal tumors and the 5‐year survival is only 9%. The expression of miRNAs in serum has been proved to be related to tumorigenesis and development of cancers. The miRNA targets and gene targets were predicted in *microRNA*.*org*, *miRDB*, *TargetScan*, and *RNAInter*. The expression data of *STK31* (*Serine*/*Threonine Kinase 31*) and miRNAs generated from PC samples was from TCGA and the relationship of expression of *STK31* and miR‐543 was confirmed in PC samples from our center. Double luciferase reporter gene assay was used to demonstrate the direct binding between miR‐543 and *STK31*. The effect of expression level of miRNAs on survival time was assessed by Kaplan–Meier curves. The Go Ontology (GO) and the Kyoto Encyclopedia of Genes and Genomes (KEGG) pathway analysis of miR‐543‐related genes were performed. The results showed that miR‐543 had a statistically significant correlation with the expression of *STK31* and contained the direct binding site with *STK31*. The expression level of miR‐543 may affect the survival of PC. The results of GO and KEGG pathway analysis showed that miR‐543 might play a key role in Insulin signaling pathway. MiR‐543 could be combined with *STK31* and affect the expression of *STK31*. The expression of miR‐543 could also predict the survival of patients with PC, which suggested that miR‐543 might play an important role in PC. The GO and KEGG pathway analysis also displayed that miR‐543 was involved in several other pathways of pancreas.

## Graphical abstract

miR‐543 has the sequence of STK31 binding site and its expression was negatively correlated with STK31 expression in PC. miR‐543 was also correlated with 1548 genes expression and the enrichment study was found that they enriched in Insulin signaling pathway, ErbB signaling pathwayn and T cell receptor signaling pathway. Our results indicate that miR‐543 may play an important role and is a promising therapeutic target in PC.

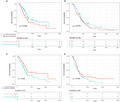

## 1 INTRODUCTION

Pancreatic cancer (PC) is one of the most malignant gastrointestinal tumors worldwide, with more than 56,770 estimated newly diagnosed cases per year in United States in 2019.^1^ As the most frequent cause of cancer deaths, PC is an extremely lethal disease with 45,750 cancer deaths annually. For all stages combined, PC has the worst prognosis with the lowest 5‐year survival (9%).^1^ Although it is well known that the serum carbohydrate antigen (CA) 19‐9, CA125, and carcinoembryonic antigen (CEA) are the most commonly molecular markers used to assist diagnosis and predict prognosis, the accuracy of the prediction is not ideal and our understanding of the mechanism is limited.^2^, ^3^, ^4^, ^5^, ^6^ Therefore, there is a great need to identify novel biomarkers to improve the prediction of prognosis of PC and explain its possible molecular mechanism.

MicroRNAs (miRNAs) are a family of small noncoding RNAs (21–23 nucleotides), which play important roles in regulating gene expression by binding imperfectly to the 3´‐untranslated region of target mRNAs.^7^, ^8^ After the first report about the role of miR‐15 and miR‐16 in chronic lymphocytic leukemia (CLL) in 2002,^9^ lots of studies have drawn the conclusion that the expression of miRNAs in plasma/serum is associated with many cancers, including PC.^9^, ^10^, ^11^, ^12^ Therefore, miRNAs may be potentially stable noninvasive biomarkers in PC. However, these miRNAs, which could definitely affect some gene expression in tissues, seem more important to the prediction of diagnosis and prognosis in cancers. Previously, study has suggested that *STK31* plays an important role in PC and we recently found that miR‐543 was related to the expression of *STK31*. It have been found that miR‐543 expression could affect tumor proliferation, invasion, and migration in several cancers.^13^, ^14^, ^15^ Thus, we hypothesize that miR‐543 is associated with the development of PC by interacting with *STK31* and it is supposed to be a promising marker for prediction of prognosis of PC.

Here, we identify the relationship between miR‐543 and *STK31* and confirm the value in aiding to predicting clinical prognosis of PC. And we also attempt to explore the potential function of miR‐543 in PC including the genetic networks of its downstream.

## 2 METHODS

### 2.1 Public data sets

TCGA public databases for pancreatic cancer (PAAD data set, released on 1 June 2015 https://tcga‐data.nci.nih.gov/tcga/tcgaHome2.jsp) ^16^ was used. It contained multiomics information including the clinical, gene, and miRNA expression data of PC, which could be used to evaluate the expression pattern of miRNAs and genes. In TCGA, 170 PC samples had gene expression, miRNA expression, and clinical information. RNA‐Seq by Expectation‐Maximization (RSEM) and Reads Per Million (RPM) was used to present the expression of genes and miRNAs from RNA‐Seq, respectively.

### 2.2 Study population

Fifty frozen tumor tissues from PC patients stored in Pancreas Biobank from the First Affiliated Hospital of Nanjing Medical University, who underwent initial surgical resection between December 2016 and April 2017 in the pancreas center of the First Affiliated Hospital of Nanjing Medical University, were tested. There was no treatment before surgery for these patients and there was a documented survival of at least 3 months from the date of surgery, which mean that we excluded those people who died from surgical complications. All patients agreed to participate in the study and were achieved informed consent. This study was approved by the ethical board of the institute of the First Affiliated Hospital of Nanjing Medical University.

### 2.3 RNA/MIRNA extraction and quantitative RT‐PCR

After tumor tissue collection, they were stored in liquid nitrogen before used. RNA and miRNA isolation were performed as described previously.^17^ Briefly, total RNA was extracted from tissues using TRIzol Reagent (Invitrogen) to denature the tissues and purified using a RNeasy Mini Kit (Qiagen). MiRNA was extracted using TRIzol LS Reagent and collected using a miRNeasy Mini Kit (Qiagen) according to the manufacturer's instructions. The *STK31* and miR‐543 expression levels in the tissue samples were normalized using *ACTIN* and U6, respectively. The reverse transcriptase reactions were performed with a TaqMan RNA RT Kit (ABI) for *STK31* and TaqMan miRNA RT Kit and stem‐loop RT primers (TIANGEN) for miR‐543 using the StepOnePlus Real‐Time System (Thermo). To confirm the relationship between *STK31* and miR‐543, SYBR Green qRT PCR was performed. The primers were listed: STK31‐F: TGTCTGGTGAGGTACATTGACT, STK31‐R: GCTCCCCAAAAAGGTTGTGC; ACTIN‐F: AGCGAGCATCCCCCAAAGTT, ACTIN‐R: GGGCACGAAGGCTCATCATT; miR‐543‐F: ACATTCGCGGTGCACTTCTT miR‐543‐R: CAGTGCGTGTCGTGGAGT and U6‐F: CTCGCTTCGGCAGCACA, U6‐F: AACGCTTCACGAATTTGCGT. All reactions were carried out in three times and it included the no‐template controls. The CT values were determined using the fixed threshold settings. To calculate the relative expression levels of the miR‐543 and *STK31*, we used a standard PCR procedure to analyze the data. The 2^−△CT^ were calculated to represent the expression levels and △CT the following formulas: △CT_STK31_ = CT_STK31_−CT_ACTIN_ for the gene *STK31* and △CT_miR‐543_ = CT_miR‐543_−CT_U6_ for miR‐543 expression.

### 2.4 MIRNA target prediction

*microRNA*.*org* (http://www.microrna.org), *miRDB* (http://www.mirdb.org/), *TargetScan* (http://www.targetscan.org/vert_72/), and *RNAInter* (http://www.rna‐society.org/raid/home.html) were used to predict the targets of miRNAs. *microRNA*.*org* is a comprehensive resource of microRNA target predictions and expression profiles and target predictions are based on miRanda algorithm which incorporates current biological knowledge on target rules and on the use of an up‐to‐date compendium of mammalian microRNAs.^18^ *miRDB* is an online database for miRNA target prediction and functional annotations which uses common features associated with miRNA binding and target downregulation to identify and predict miRNA targets with machine learning methods.^19^, ^20^ *TargetScan* predicts biological targets of miRNAs by searching for the presence of conserved 8mer, 7mer, and 6mer sites which match the seed region of each miRNA.^21^, ^22^ *STK31* was performed as targeted gene being regulated and miR‐543 as miRNA playing the regulating function. *RNAInter* is a comprehensive resource for RNA interactome data obtained from the literature and other databases, containing over 41 million RNA‐associated interactions of RCI, RDI, RHI, RPI, and RRI ^23^.

### 2.5 Dual‐luciferase reporter assays

A dual‐luciferase reporter assay was used to assess the direct binding site between STK31 and miR‐543. To construct the STK31 promoter luciferase vector, sequences (730 bp) include the wild‐type 3′‐untranslated region (UTR) of STK31 and ACTIN were synthesized by GeneBay (Nanjing) and subcloned into the BsiWI‐XhoI restrictive sites of the pEZX‐FR02 Vector. 293T cells were co‐transfected with the pEZX‐FR02 reporter vectors and miR‐543 mimic using Lipofectamine 2000 reagent (Thermo Fisher Scientific, Inc.). After 48 h incubation at 37°C with 5% of CO2, luciferase activity was evaluated. Firefly luciferase activity was normalized to Renilla luciferase (Promega Corporation) gene activity with a dual‐luciferase reporter assay (Promega Corporation). miR‐543 mimics (5′‐AAACAUUCGCGGUGCACUUCUU‐3′) and its negative control (NC mimics; 5′‐UUCUCCGAACGUGUCACGUTT‐3′) were synthesized by Guangzhou RiboBio Co., Ltd. This reporter assay was performed three times.

### 2.6 Statistical analysis

Spearman correlation analysis was performed to establish the relationship between all miRNAs and *STK31* expression to find these potentially functional miRNAs both in the data from our center and TCGA database. Data of functional experiments were assayed by comparing mean ±SD using a unpaired Student's *t*‐test for independent two groups and one‐way ANOVA followed by Tukey's test for independent multiple groups and were assumed for *p < 0.05, **p < 0.001, ***p < 0.0001. Kaplan–Meier curves were performed to evaluate the prognostic value of miRNAs and *STK31* in PC, and log‐rank testing was used to state the statistical significance. When we focused on the miR‐543, the relationship between all genes (20,532 genes, including *STK31*) with miR‐543 was analyzed also by spearman correlation test (*p* < 1 × 10^−6^). The Gene Ontology (GO) knowledgebase (http://www.geneontology.org/) is the largest source to provide structured and well‐established vocabulary for annotating proteins from cellular component (CC), biological process (BP), and molecular function (MF) aspects^24^, ^25^. The Kyoto Encyclopedia of Genes and Genomes (KEGG) (http://www.genome.ad.jp/kegg/) is a database resource for understanding high‐level functions and utilities of the biological system and predicting the potential pathways for genes.^26^, ^27^ The R package “clusterProfiler (v3.12.0)”^28^ was used to analyze the GO term and KEGG pathway for these miR‐543‐related genes and dotplot was used to present the identified GO term and KEGG pathway. The significant threshold of these enrichment analysis was FDR <0.05. All of the statistical analyses were performed with R software (version 3.6.3) and *p* < 0.05 was considered as statistical significance for all the tests.

## 3 RESULTS

### 3.1 Prediction of *STK31*‐associated miRNAs

**FIGURE 1:**
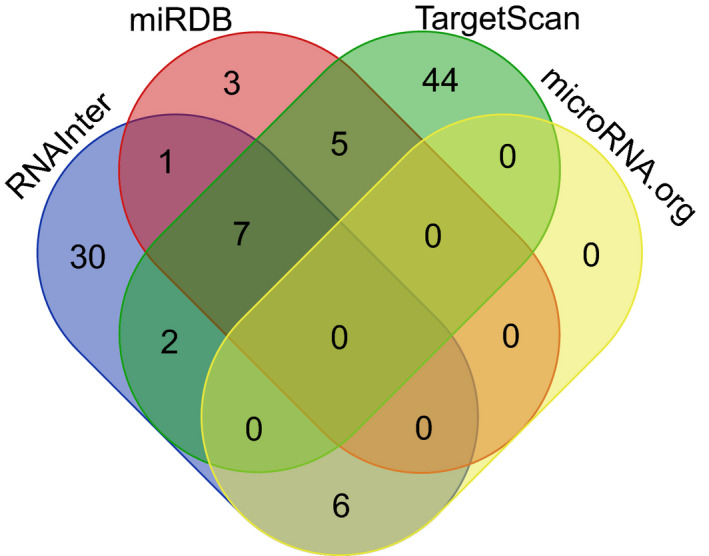
Venn diagram for predicted STK31‐associated miRNA in four database

In the databases of *microRNA*.*org*, *TargetScan*, *RNAInter*, and *miRDB*, 6 miRNAs, 58 miRNAs, 46 miRNAs, and 16 miRNAs were predicted to correlate with *STK31* in Homo sapiens, Mus musculus, or Rattus norvegicus. Since the prediction principles of these databases are different, 21 miRNAs, which we selected, appeared in at least two databases (*RNAInte*r, *TargetScan*, and *miRDB*: miR‐8084, miR‐7243, miR‐3140, miR‐561, miR‐4772, miR‐589, miR‐1912; *RNAInter* and *miRDB*: miR‐196c; *RNAInter* and *TargetScan*: miR‐153, miR‐3120; *RNAInter* and *microRNA*.*org*: miR‐490, miR‐300, miR‐381, miR‐543, miR‐495, miR‐186; *TargetScan* and *miRDB*: miR‐223, miR‐4777, miR‐3145, miR‐335, miR‐6417.) and these miRNAs were studied by expression correlations with *STK31* in TCGA (Figure 1).

### 3.2 Expression correlations between *STK31* and MIRNAS in TCGA

**FIGURE 2:**
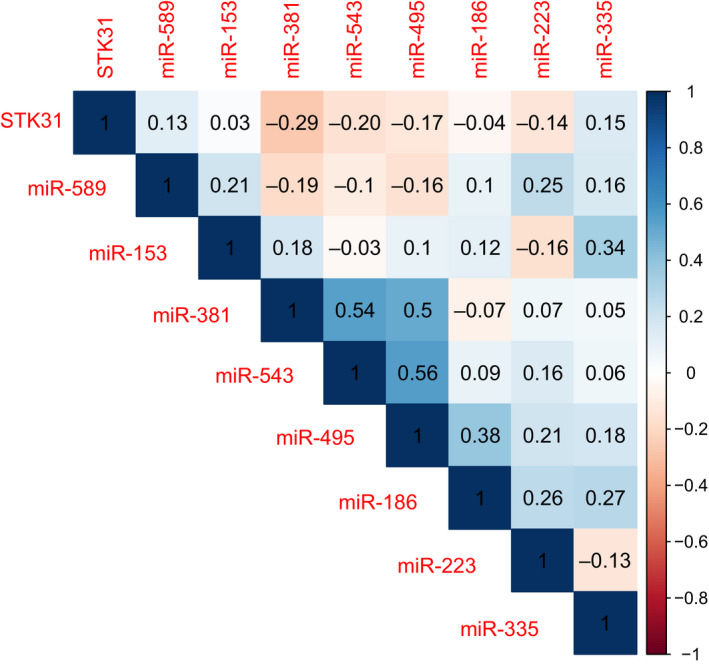
The expression correlation between the expression of *STK31* and 10 miRNAs in TCGA

From TCGA database, the expression level of *STK31* and all these 15 of 21 miRNAs predicted by Public Database was acquired. The other six miRNAs (miR‐8084, miR‐7243, miR‐4772, miR‐196c, miR‐4777, and miR‐6417) were not contained in TCGA miRNA database. All these eight miRNAs, whose median reads per million (RPM) was greater than 1, were then selected to analyze the expression correlation with the expression of *STK31* (Figure 2). Three miRNAs (miR‐543, miR‐495, and miR‐381) were significantly correlative with *STK31* (*R* = −0.20, spearman *p* = 0.0076; *R* = −0.17, *p* = 0.0207; *R* = −0.29, *p* < 0.0001, respectively).

### 3.3 Relationship between MIR‐543 and prognosis of PC

**FIGURE 3:**
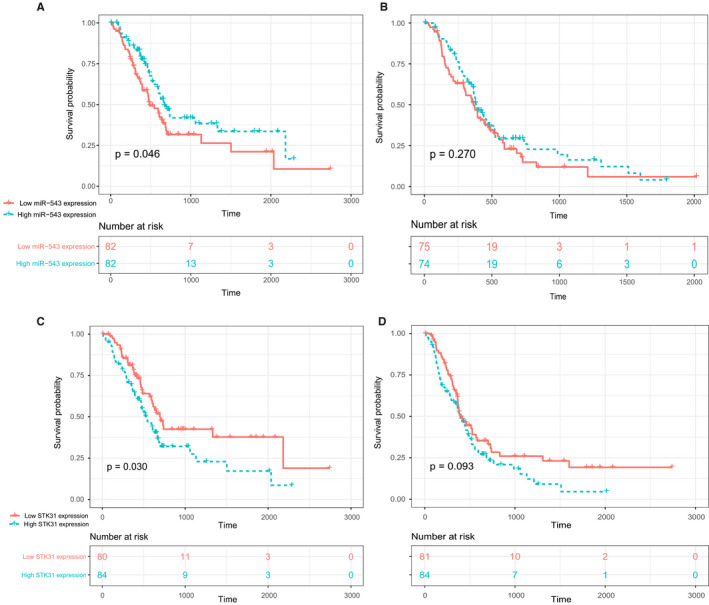
Overall survival (A) and disease‐free survival (B) of PC in low‐ and high‐miR‐543 expression groups and OS (C) and disease‐free survival (D) of PC in low‐ and high‐*STK31* expression groups in TCGA data set

After the expression correlation analysis, the prognostic correlation analysis was performed to assess the relationship between their expression and prognosis. The results showed that the high expression of miR‐543 suggested the lower overall survival (MST 485 days in high expression of *SKT31* and 666 days in low, log‐rank *p* = 0.046, Figure 3A). But, different expression levels of miR‐543 could not predict the disease‐free survival (log‐rank *p* = 0.270, Figure 3B). In contrast to miR‐543, high expression of STK31 suggested the higher overall survival (log‐rank *p* = 0.030, Figure 3C) but not in disease‐free survival (log‐rank *p* = 0.093, Figure 3D).

### 3.4 Validate the expression correlations between *STK31* and MIR‐543

**FIGURE 4:**
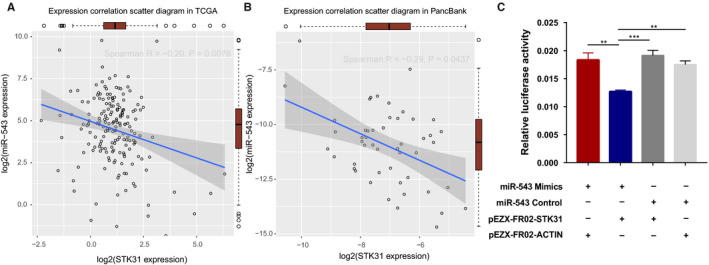
The relationship between *STK31* and miR‐543. A, Expression correlation scatter diagram in TCGA and the red box present the expression of STK31 and miR‐543. B, Expression correlation scatter diagram in our Pancreas Biobank and the red box present the expression of STK31 and miR‐543. C, Reporter gene assays with constructs containing the STK31 promoter in 293T cell lines. miR‐543 control or mimics was co‐transfected into cells. Data shown are means ± SD

In addition to the analysis in TCGA database, we also confirmed the relationship between the expression of miR‐543 and gene *STK31* in 50 pancreatic cancer tissues from our Pancreas Biobank. The basic characteristics of these patients are shown in Table 1. The results were consistent with the TCGA database and they confirmed that miR‐543 expression was negatively correlated with *STK31* expression (Spearman *R* = −0.28, *p* = 0.0437, Figure 4A,B). With dual‐luciferase reporter assays, we found that the miR‐543 mimics co‐transfected with pEZX‐FR02‐STK31 significantly decreased the promoter activity of the reporter gene, leading to lower *STK31* expression (Figure 4C).

**TABLE 1:** Clinical characteristics of patients with PC from our Pancreas Biobank

	Characteristic
Age (y, Mean ± SD)	61.7 ± 10.8
Gender N(%)	
Male	28 (56)
Female	22 (44)
CA19‐9 (U/ml, IQR)	173（53–524）
CEA (U/ml, IQR)	3.1（1.7–5.3）
T stage N(%)	
T1	5 (10)
T2	33 (66)
T3	4 (8)
T4	5 (10)
Unknown	3 (6)
N stage N(%)	
N0	25 (50)
N1/N2	25 (50)
M stage N(%)	
M0	50 (100)
M1	0 (0)
TNM stage N(%)	
I	17 (34)
II	19 (38)
III	11 (22)
Unknown	3 (6)
Grade N(%)	
I/I–II/II	11 (22)
II–III/III	37 (74)
Unknown	2 (4)

### 3.5 The functional and pathway enrichment analysis of MIR‐543‐related genes

**FIGURE 5:**
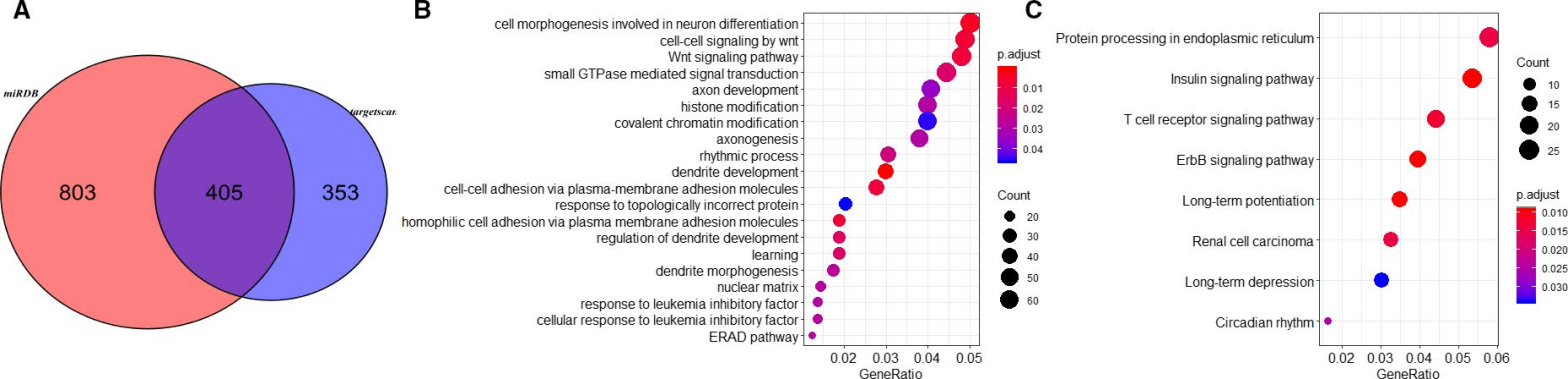
The functional and pathway enrichment analysis of miR‐543‐related genes. A, Venn Diagram for the predicted the targets of miR‐543 in *miRDB* and *TargetScanHuman*. B, GO term enrichment of miR‐543‐related genes. C, KEGG enrichment of miR‐543‐related genes

We then predicted the targets of miR‐543 in *miRDB* and *TargetScanHuman*. In all, 1208 and 758 genes were identified as potential target genes of miR‐543. After removing the represented genes, 1548 genes were left (Figure 5A, Table S1). Based on the GO term and KEGG analysis, a total of 19 GO_BP terms, 27 GO_CC terms, 19 GO_MF terms, and 13 KEGG pathways were enriched for the miR‐543‐related genes. The GO terms included “dendrite development” (BP GO:0016358; FDR, 0.0002841753), “ubiquitin‐like protein transferase activity” (MF GO:0019787; FDR, 0.002539626), and “protein serine/threonine phosphatase activity” (MF GO:0004722; FDR, 0.011598712) (Figure 5B). In addition, significant pathways included “Insulin signaling pathway” (FDR, 0.00919), “ErbB signaling pathway” (FDR, 0.00919) and “T cell receptor signaling pathway” (FDR, 0.012167) (Figure 5C).

## 4 DISCUSSION

A great deal of evidence suggests that miRNAs are stable diagnostic and prognostic biomarkers for cancers. Previously, several studies have been related to miR‐543. Dysregulation of miR‐543 expression could influence tumor proliferation, invasion, and even migration in colorectal cancer,^14^ hepatocellular carcinoma, clear cell renal cell carcinoma^13^ cervical cancer,^29^ and gastric cancer.^30^ But the results of these studies are not completely consistent in different cancers. Sun et al. demonstrated that they identified miR‐543 as tumor promoter and plays a vital role in CRC metastasis by direct targeting PTEN directly.^14^ Yu et al. also demonstrated that miR‐543 was a tumor promoter and dramatically overexpressed in hepatocellular carcinoma (HCC). The overexpression of miR‐543 could promote the ability of proliferation and invasion in HCC cell line by targeting PAQR3.^15^ Moreover, miR‐543 promoted cell proliferation by targeting SIRT1^31^ and cell migration and invasion by targeting sPOP^30^ in gastric cancer. But Li and his team came to the conclusion that miR‐543 was decreased in endometrial cancer and suppressed endometrial cancer oncogenicity via targeting FAK and TWIST1^32^ and XU et al. reported that miR‐543 might function as a tumor suppressor in patients with glioma.^33^ The different function of miR‐543 in different types of tumor suggested that miR‐543 might play different roles in different cancers. Up to now, there is no report about miR‐543 in PC has been published. Therefore, we investigated the possible role of miR‐543 in PC.

Based on the previous study of *STK31*,^34^ we further studied its related miRNAs in current study and found a novel potentially important miRNAs, miR‐543, in PC. First, we searched in predictions tools for *STK31*‐related miRNAs. After we identified *STK31*‐related miRNAs, we analyzed the relationship between these miRNAs and the survival time of PC in TCGA database. The results showed that miR‐543 was significantly related with the prognosis of PC. In addition, we confirmed the expression correlationship between miR‐543 and *STK31* in PC tissue specimen. At last, we predicted the miR‐543‐related genes and uncovered that miR‐543 might play roles in pathways of insulin signaling, ErbB signaling pathway, and T cell receptor signaling in PC.

Previous studies have strongly suggested that miR‐543 can regulate cell invasion and impedes apoptosis by activation of the Wnt/β‐catenin pathway though Smad7^35^ and promotes cell proliferation and metastasis through the Wnt/β‐catenin pathway by targeting Dickkopf 1 in cancers.^36^ These results are consistent with our findings in bioinformatic analysis in PC. We also found that cell–cell signaling by Wnt and Wnt signaling pathway was enriched in miR‐543‐related genes. Moreover, researches have confirmed that miR‐543 is involved in regulating cell invasion and metastasis in renal cell carcinoma. And in this study, cell–cell adhesion via plasma‐membrane adhesion molecules in GO_BP and renal cell carcinoma in KEGG were also enriched. Neamah WH et al.^37^ studied the aryl hydrocarbon receptor (AhR) and found that miR‐543 could target these anti‐inflammatory, which was consistent with our findings of the relationship between miR‐543 and T cell receptor signaling pathway.

In previous studies, we have displayed that *STK31* expression was significantly higher in patients with poorer prognosis, suggesting that *STK31* had potential clinical value.^34^ And we also found that *STK31* was reactivated by demethylation. As one of the common mechanisms regulating gene expression, we predicted that the expression of *STK31* was regulated by miR‐543 and had the sequence of miR‐543 binding. We had confirmed their expression correlationship in PC tissues by qPCR. *STK31* is one of miR‐543‐regulated genes and miR‐543 may play an important role in PC. And we also confirmed the direct binding between miR‐543 and STK31 by dual‐luciferase reporter assays. Despite the exciting probability of therapeutic targets in PC, there are also some challenges existed. The major one is that miRNA‐based therapeutics can cause unexpected side effects because miRNAs have more than one downstream‐regulated gene and it is difficult to ensure tumor‐specific delivery and retention of miRNAs. Therefore, off‐target effects are likely to be happened.^38^ To overcome this shortcoming, we need to carry out adequate animal and clinical trials. Another limitation is that miRNAs inhibitors are not stable in vivo.^38^ MiRNA introduced into mice via the tail vein will be cleared in 30 mins from the circulatory system^39^. We need to improve existing methods to make inhibitors more stable. Once we could solve these problems, miRNA must be a very meaningful therapeutic targets.

In conclusion, this study showed that miR‐543 can directly binding with *STK31* and its expression was negatively correlated with *STK31* expression in PC. In addition to the relationship with *STK31*, miR‐543 was also correlated with 1548 genes expression and the enrichment study was found that they enriched in several KEGG pathway, such as Insulin signaling pathway, ErbB signaling pathway, and T cell receptor signaling pathway, which provided comprehensive insight into the potential molecular mechanisms in PC. Taken together, our results indicate that miR‐543 may play an important role and is a promising therapeutic target in PC.

## CONFLICTS OF INTEREST

All authors have reviewed the final version of the manuscript and approved it for publication. The authors have no conflicts of interest to declare.

## AUTHOR'S CONTRIBUTIONS

Weizhong Yuan & Hao Gao: They contributed equally to this work. They were mainly responsible for acquisition of data, analysis and interpretation of data, and drafting the article. Guangfu Wang: He provided assistance for data acquisition and statistical analysis. Yi Miao & Kuirong Jiang: They were responsible for revising it critically for important intellectual content. Kai Zhang & Junli Wu: They contributed to the conception and design of the study and coordinate the work of all parties. Kai Zhang was also responsible for the verification of data and analytical methods; Junli Wu also contributed to the final approval of the version to be submitted.

## Supporting information

Table S1

## Data Availability

Some or all data generated or used during the study are available from the corresponding author by request.
